# Differently Environment Stable Bio-Silver Nanoparticles: Study on Their Optical Enhancing and Antibacterial Properties

**DOI:** 10.1371/journal.pone.0077043

**Published:** 2013-10-09

**Authors:** Yekkuni L. Balachandran, Shanmugam Girija, Rajendran Selvakumar, Saowanit Tongpim, Arno C. Gutleb, Sarvajeyakesavalu Suriyanarayanan

**Affiliations:** 1 Department of Biotechnology, Bharathiar University, Coimbatore, India; 2 Department of Nanobiotechnolgy, PSG Institute of Advanced Studies, Coimbatore, India; 3 Department of Microbiology, Khon Kaen University, Khon Kaen, Thailand; 4 Department of the EnvironmentandAgrobiotechnologies, Centre de Recherche Public-Gabriel Lippmann, Belvaux, Luxembourg; 5 Department of Water and Health, JSS University, Mysore, Karnataka, India; King's College London, United Kingdom

## Abstract

Generally, limited research is extended in studying stability and applicational properties of silver nanoparticles (Ag NPs) synthesized by adopting ‘green chemistry’ protocol. In this work, we report on the synthesis of stable Ag NPs using plant-derived materials such as leaf extract of Neem (*Azadirachta indica*) and biopolymer pectin from apple peel. In addition, the applicational properties of Ag NPs such as surface-enhanced Raman scattering (SERS) and antibacterial efficiencies were also investigated. As-synthesized nanoparticles (NPs) were characterized using various instrumentation techniques. Both the plant materials (leaf extract and biopolymer) favored the synthesis of well-defined NPs capped with biomaterials. The NPs were spherical in shape with an average particle size between 14-27 nm. These bio-NPs exhibited colloidal stability in most of the suspended solutions such as water, electrolyte solutions (NaCl; NaNO_3_), biological solution (bovine serum albumin), and in different pH solutions (pH 7; 9) for a reasonable time period of 120 hrs. Both the bio-NPs were observed to be SERS active through displaying intrinsic SERS signals of the Raman probe molecule (Nile blue A). The NPs were effective against the *Escherichia coli* bacterium when tested in nutrient broth and agar medium. Scanning and high-resolution transmission electron microscopy (SEM and HRTEM) images confirmed cellular membrane damage of nanoparticle treated *E. coli* cells. These environmental friendly template Ag NPs can be used as an antimicrobial agent and also for SERS based analytical applications.

## Introduction

Metallic silver nanoparticles (Ag NPs) have gathered much attention due to their unique properties, which depend on their morphology, dimension and colloidal stability. Colloidal stability of the nanoparticles (NPs) in solvent other than water is desired in most of the biological applications and in several other analytical applications [1]. Hence, understanding the stability of Ag NPs in different environment is essential, which likely exhibits the fate of the NPs. Colloidal stability is a function of many factors including the type of capping agent, surrounding environmental conditions like pH, ionic strength and the background electrolyte composition [2,3]. Unstable nanoparticle (NP) aggregates may significantly contribute to dissolution of ions from NPs [4], which increases during particle storage [5]. Such coexistence of the NP and its ionic forms may induce toxic pathway [6,7]. The colloidal stability of the NPs is crucial, which determines their mobility, bioavailability and toxicity in any ecosystem [8,9]. Hence it is essential to synthesis stable NPs, which can minimize ion dissolution and retain its physicochemical properties.

The capping molecules bound to NP surface are the ones that give definite shape, size and stability to the NPs. Of the various synthesis methods, most of the stable NP syntheses have been achieved through wet chemical methods. Due to the growing interest in green chemistry methods in recent years, biological and polymer based synthesis of NPs has gained significant interest [10,11]. However, most of the green chemistry approaches for NP synthesis from sources such as microbial, plant extracts and polymers have stopped at the level of synthesis and basic characterization. Considering the wide application of NPs in various fields, the study on the stability and application of the NPs synthesized by ‘green chemistry’ has become very essential.

In the present work, we have used leaf extract of Neem (*Azadirachta indica*) and biopolymer pectin from apple peel for synthesis of Ag NPs. Polymers as well as the essential components in plant leaf extract such as proteins and sugars are known to provide stability to the NPs. The colloidal stability of Ag NPs in different electrolyte medium (NaCl and NaNO_3_), biological medium (bovine serum albumin [BSA]) and in different pH solutions (pH 7 and 9) was determined by measuring the changes in UV-Vis absorbance spectra [1], hydrodynamic diameter (HDD) and zeta potential value. In addition, the NPs were studied for their optical field enhancing properties through surface-enhanced Raman scattering (SERS) of Raman probe molecule Nile blue A (NBA), and antibacterial activity using *Escherichia coli* as the model organism. *E. coli* has been a major fecal coliform contaminant and, an indicator organism in water and also *E. coli* causes major foodborne diseases; hence it has been used as the model organism in the present study.

## Materials and Methods

### Materials

**Figure 1:**
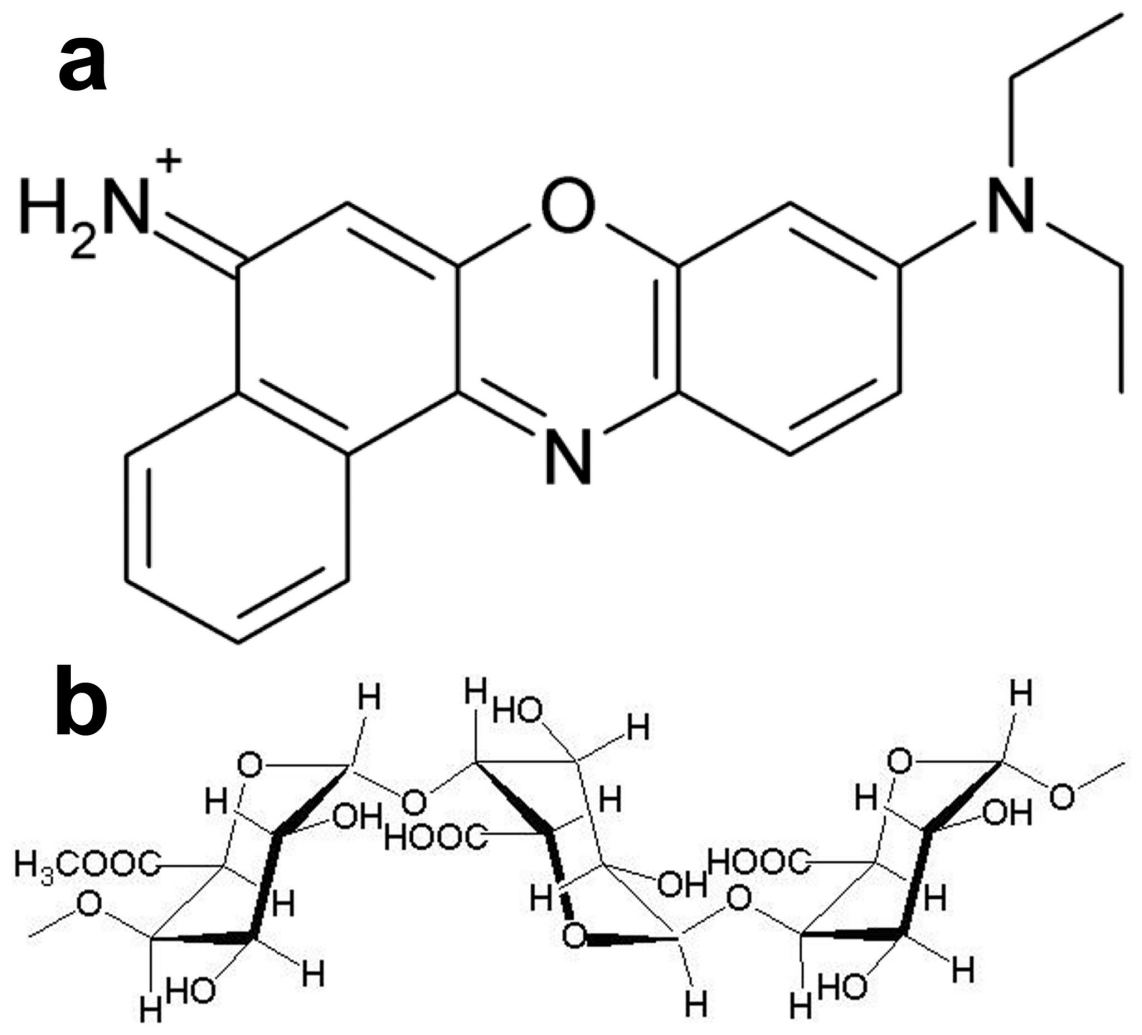
Molecular structure of biopolymer pectin and Raman analyte Nile Blue A. Structure of (a) Nile blue A and (b) pectin.

AgNO_3_, pectin (from apple peel), NBA, NaCl and NaNO_3_ were purchased from Sigma-Aldrich (Bangalore, India) and used as received. Molecular structure of NBA and pectin are shown in Figure 1. BSA and nutrient broth (NB) were purchased from HiMedia chemicals India Pvt. Ltd (Mumbai, India). Ultra pure water with a resistivity more than 18.0 MΩ (Millipore Milli-Q system) was used for preparation of all aqueous solutions. All the glasswares were cleaned with freshly prepared aqua regia (3:1, HCl:HNO_3_) and rinsed thoroughly with water. Then they were dry sterilized using hot air oven at 160 °C for 3 hrs, prior to use.

### Silver Nanoparticles Preparation and Characterization

#### Preparation of silver nanoparticles using plant leaf extract

*Azadirachta indica* leaves were collected freshly and washed thoroughly with sterile Milli-Q grade water to remove adsorbed impurities. 5 g of finely chopped leaves were boiled (~ 80 °C) with 50 mL of water for about 10 minutes and then brought to room temperature (~ 27 °C). The filtrate was then separated using Whatman grade I filter paper and stored at 4 °C until further use.

In a typical experiment, 1 mL of 0.1 M aqueous AgNO_3_ was added to 99 mL of aqueous leaf extract solution (10% leaf extract in final working solution) under gentle stirring. The pH of the working solution was adjusted to 7 using 0.5 M NaOH. The reaction was allowed to proceed under gentle stirring at room temperature for 12 hrs. The NPs were separated by centrifugation at 10,000 × g for 20 minutes at 20 °C and washed thrice with water to remove the impurities. Finally the NPs were filtered and concentrated using centrifugal filter devices (Amicon Ultra- 15 3K Millipore). The Ag NPs were stored at 4 °C before being used for further analysis.

#### Preparation of silver nanoparticles using biopolymer pectin

Aqueous biopolymeric solution containing 10 g/L of pectin (from apple peel) was prepared by placing the mixture in warm water bath (~ 60 °C) for about 15 min for complete solubilization and then brought down to room temperature. To the biopolymeric solution, aqueous 0.1 M AgNO_3_ (1 mM final concentration) and NaOH solutions (final concentration 25 mM) were added rapidly under vigorous stirring. After 6 hrs of stirring at room temperature, the NPs were separated and washed thrice with water by centrifugation at 12,000 × g for 25 minutes at 25 °C. Finally the NPs were filtered and concentrated using centrifugal filter devices (Amicon Ultra- 15 3K Millipore). The concentrated stock colloidal NPs were stored at 4 °C.

#### Characterization of nanoparticles

UV-Vis absorption spectroscopy was performed on a PerkinElmer (LAMBDA 35 Singapore) UV/Vis spectrophotometer operating at 1 nm resolution. The size and morphology of NPs were examined using high-resolution transmission electron microscopy (HRTEM) JEOL JEM 2100 (Japan) and Philips CM20 TEM (Netherlands) operating at 200 kV. The samples were prepared by placing a drop of NPs on a carbon coated copper grid. The chemical analysis of the NPs was performed using energy dispersive X-ray spectroscopy (EDS) incorporated to the TEM. Powder X-ray diffraction (PXRD) measurements of Ag NPs were performed on a PANalytical X-ray diffractometer (XPERT PRO, Netherlands) using monochromatic Cu Kα radiation. Fourier transform infrared (FTIR) analysis was carried out using JASCO FT/IR 4100 (Japan) Fourier transform infrared spectrometer. The samples were recorded with KBr, at a spectral range 4000-400 cm^-1^, scanning speed of 2 mm sec^-1^ and resolution at 4 cm^-1^. DLS and zeta (ζ) potential measurements of NPs were performed with a zetasizer (Malvern Zetasizer Nano ZS90, UK) at room temperature.

### Colloidal Stability of Silver Nanoparticles

For all stability experiments, a small volume of NP stock suspension was diluted with respective solutions. The stability of NP was determined in media such as water, NaCl and NaNO_3_ (electrolyte) solutions of concentrations 50 and 200 mM, 0.1 and 0.25% BSA (biological) solutions, and in pH 7 and 9. A precalculated amount of electrolyte solution or biological solution was added to the NP suspension to obtain the desired concentration making the final suspension volume of 50 mL. For pH stability, a small amount of NP stock solution was dispersed in 50 mL solutions of different pH (pH 7 and 9). The initial measurements were started almost immediately (0.5 hrs), and the changes in the UV-Vis absorbance spectra, HDD and electrophoretic mobility (surface charge) were recorded over 5 days at different time intervals. Small amount of the samples were retracted for analysis at different interval. All the analysis were conducted at a temperature of 25 ± 2 °C and the samples were stored at 25 ± 2 °C in dark, in an airtight container over the period of study.

### SERS Activity of Silver Nanoparticles

For SERS experiments, 50 µl of the Ag colloid was mixed with 50 µl of the Raman analyte NBA solution (1 × 10^−6^ M final concentration), and then made up to a final volume of 0.3 mL using ultrapure water followed by vigorous mixing. The analyte-NPs mixture was dispersed thoroughly in water by sonication. SERS spectra for the samples were acquired using a PerkinElmer RamanFlex^TM^ 400 Fibre Optic Analyzer (USA) at 532 nm at a power of ~ 32 mW. Spectra were accumulated for 1 second. Each spectrum was the average of 5 scans.

### Antibacterial Activity of Silver Nanoparticles

#### Organism

*E. coli* was grown overnight in NB at 37 °C. Cells were diluted in NB, and optical density (OD) at 600 nm (OD_600_) was adjusted to 0.1 (OD of 0.1 corresponds to a concentration of 10^8^ colony forming units [CFU]/mL at 600 nm).

#### Bacterial susceptibility to silver nanoparticles

To examine the bacterial susceptibility assay, the *E. coli* cells were cultured on a nutrient agar plate supplemented with Ag NPs (10-60 μg/ plate). Approximately 10^5^ CFU of *E. coli* were cultured on nutrient agar plates supplemented with Ag NPs. The plates were incubated at 37 °C for 24 hrs and the number of colonies were manually counted [12,13]. Ag NPs free nutrient agar plates and plates supplemented with antibiotic (ampicillin; 10-60 µg) grown under same conditions were used for comparison.

#### Bacterial growth in the presence of silver nanoparticles

To study the bacterial growth rate in presence or absence of Ag NPs and antibiotic (ampicillin), *E. coli* were grown in 100 mL of NB medium supplemented with different doses of Ag NPs (10-60 µg) and ampicillin (10-60 µg) at 37 °C under continuous stirring on an orbital shaker. Growth rate was determined by measuring OD_600_ at various times. OD values were converted into CFU/ mL [12,13].

#### Electron microscopy analysis of Ag NPs treated bacteria

*E. coli* were grown in 20 mL NB medium overnight at 37 °C. 1 ml of 10^5^ *E. coli* cells were inoculated into 100 mL fresh NB medium containing 20 µg of Ag NPs. The bacterial suspension was collected after 3 hrs of exposure to NPs and used for scanning electron microscopy (SEM) and HRTEM analysis.

For SEM sample preparation, 20 µL of NPs treated and untreated *E. coli* cells were each deposited on polycarbonate membrane and fixed in 2.5% (v/v) glutaraldehyde in 0.1 M potassium phosphate buffer (pH 7.2). These samples were dehydrated in a series of ethanol (35, 50, 70, 80, 90, 95 and 100% for 10 min each). After drying, the samples were mounted on aluminum stubs, sputter-coated with a layer of gold and viewed under SEM (LEO 1450VP, UK) operated at 10 kV.

For HRTEM, 10 mL of NPs treated and untreated bacteria were separated by centrifugation and washed thrice with sterile water. The washed bacterial isolates were stained with 0.2% phosphotungstic acid for 5 min. The stained cells were loaded onto 3 mm copper TEM holding grid and viewed under HRTEM (JEOL JEM 2100, Japan) operated at 80 kV.

## Results

### Silver Nanoparticles Characterization

**Figure 2:**
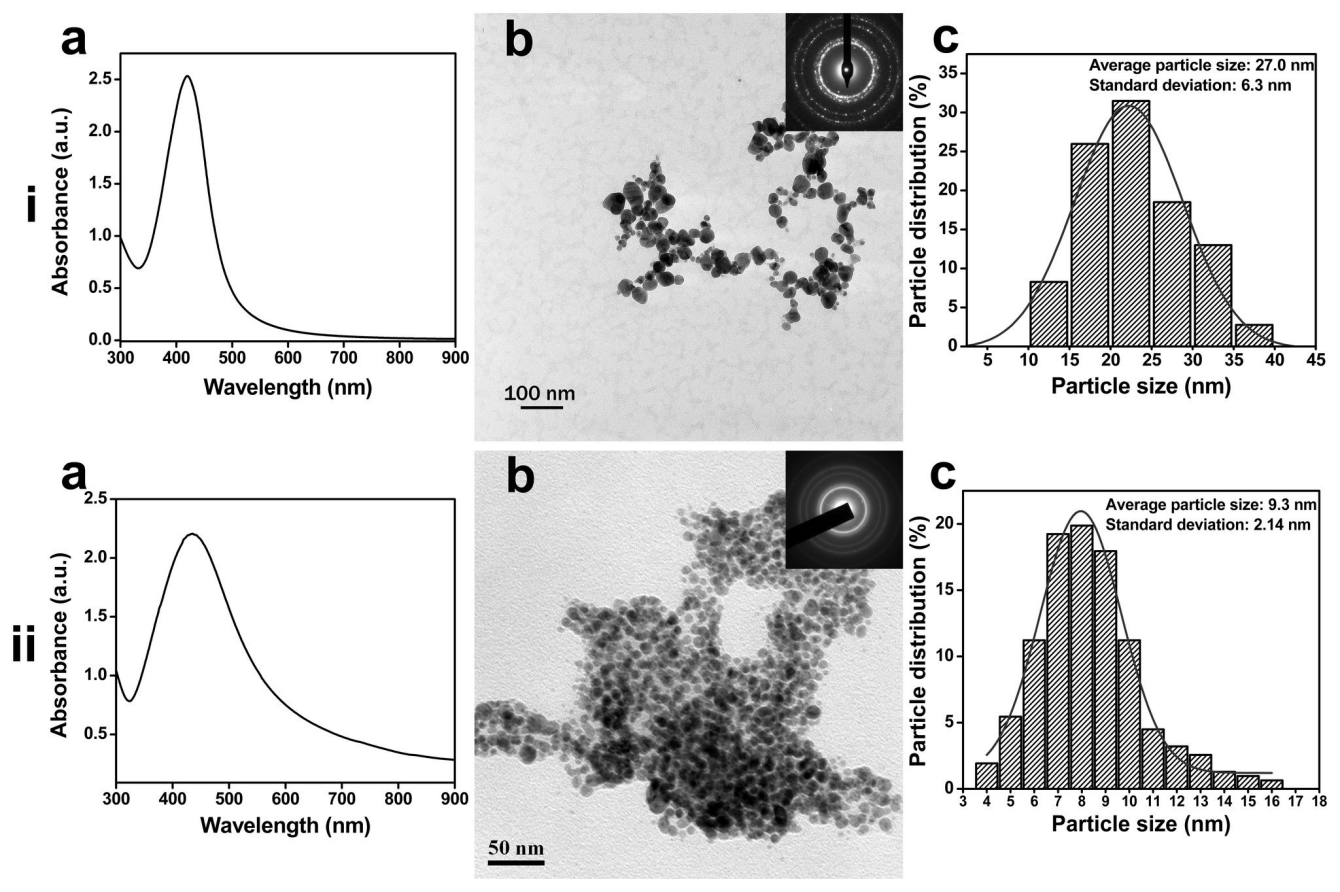
Silver nanoparticles characterization. (a) UV-Vis absorbance spectrum, (b) TEM image (inset SAED pattern) and (c) size distribution histogram pattern of (i) *A. indica* Ag NPs and (ii) pectin Ag NPs.

Ag NPs synthesized using *A. indica* leaf extract solution and biopolymer pectin were characterized using UV-Vis absorbance spectroscopy and TEM for optical and structural properties (Figure 2). *A. indica* and pectin Ag NPs showed absorption surface plasmon resonance (SPR) at 418 (Figure 2ia) and 434 nm (Figure 2iia), respectively. The morphology and dimension of the Ag NPs were determined using TEM (Figure 2ib and 2iib). From TEM images, the particles were observed to be almost spherical in shape. Inset in the TEM images are the corresponding selected area electron diffraction (SAED) patterns of the Ag NPs. The polycrystalline rings with plane distance around 2.36 Å, 2.04 Å, 1.45 Å and 1.23 Å (from inner to outer) can be assigned to the plane families (111), (200), (220) and (311) of pure fcc silver structure (see Figure S1 for corresponding powder XRD patterns). The average particle size measured by TEM was 27.0 (Figure 2ic) and 9.3 nm (Figure 2iic) for *A. indica* and pectin Ag NPs, respectively. However, the average HDD values for *A. indica* and pectin Ag NPs measured in water by DLS differed from their average diameters measured by TEM and were observed to be 33.42 and 199.2 nm (Table 1), respectively. This discrepancy may be attributed to the DLS measurement technique. In DLS, the HDD of NP include Ag core, capping agents and layers of the solvent molecules that are associated with the particle [14] while from the TEM image only the NP core size was scaled. One inherent limitation of the DLS method is taken into account; since the particle size is correlated with the scattered light intensity, presence of fewer larger diameter or aggregated NPs will dominate the intensity [15]. Both the *A. indica* and pectin Ag NPs had a negative ζ potential of −27.7 and −36.2 mV (Table 1), respectively in water, which were placed in the stable region (without aggregation) as noted in Derjaguin-Verwey Lundau-Overbeek (DVLO) theory [16,17]. Presence of elemental silver in the final NP product was evident by the EDS spectra (Figure S2).

**Table 1:** TEM primary average size, HDD and ζ potential value (in water) of Ag NPs.

**Nanoparticles**	**TEM primary size (nm)**	**HDD in water (nm)**	**ζ value in water (mV)**
*A. indica* Ag NPs	27.0 ± 6.3	33.42	−27.7
Pectin Ag NPs	9.3 ± 2.4	199.2	−36.2

**Figure 3:**
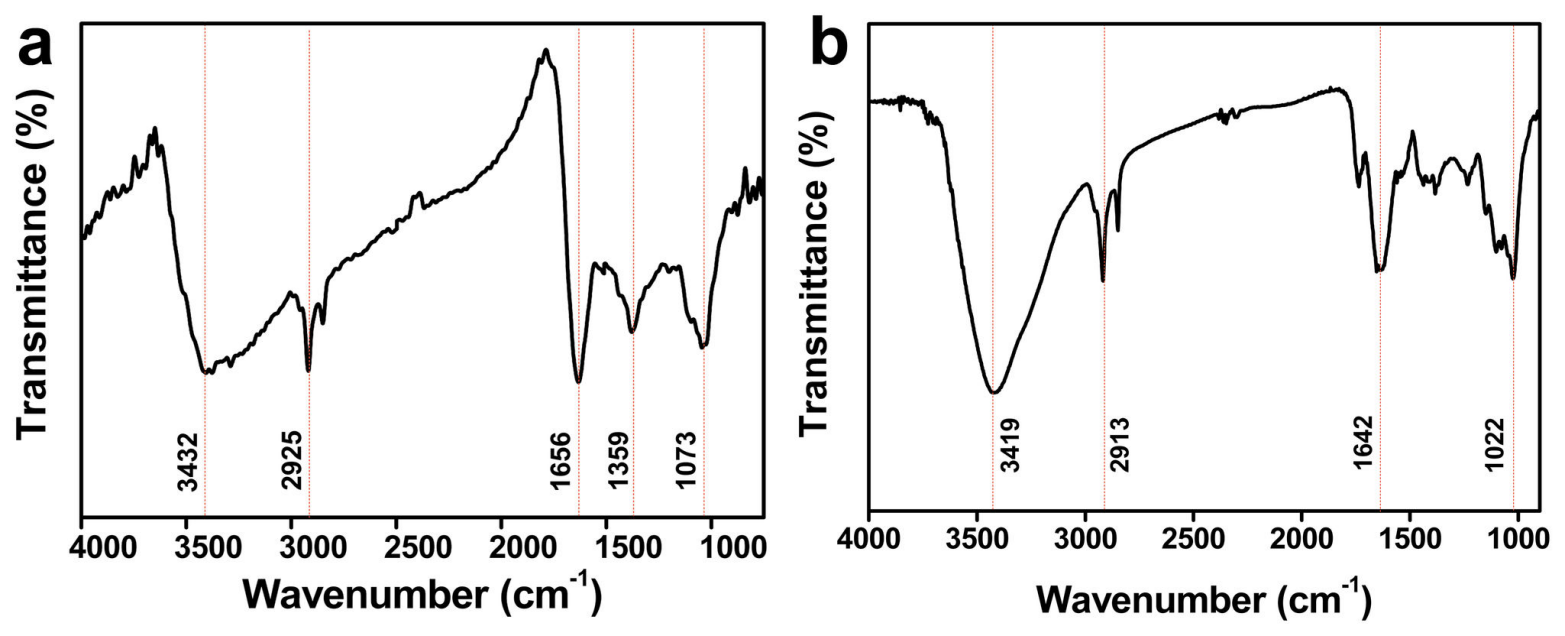
Surface composition of bio-silver nanoparticles. FTIR spectra of (a) *A. indica* and (b) pectin Ag NPs.

The surface functional groups present in *A. indica* and pectin Ag NPs were analyzed using FTIR (Figure 3). FTIR spectra of *A. indica* Ag NPs (Figure 3a) was dominated by IR peaks positioned at 3432 cm^−1^, 2925 cm^−1^, 1656 cm^−1^, 1359 cm^−1^ and 1073 cm^−1^ which correspond to functional groups of OH stretch; H−bonding of phenolic compounds, stretching modes of aldehydic C–H, stretching vibration of amide ν(C=O) of proteins, bending vibrations of geminal methyl or symmetric stretching of carbonyl group, and antisymmetric stretching of C–O group of polysaccharide and/or chlorophyll, respectively. Presence of these functional groups confirms the presence of bio-molecules on NPs’ surface, which may depict proteins, polyphenols, flavonoids, and carbohydrates present in the leaf extracts (see Figure S3 for leaf extract characterization).

Figure 3b shows the FTIR spectra of pectin Ag NPs. It was observed from the IR spectra of pectin Ag NPs that most of the peaks appear at almost the same wavelength as seen for pure pectin (Figure S4). The peak at 1022 cm^−1^ can be attributed to C=O or C=C group of pectin. Stretching vibration at 1642 cm^−1^ is related to COO^−^ group of pectin. The peak at about 2913 cm^−1^ arises from CH_2_ group and peak positioned at 3419 cm^−1^ can be indexed to ν(OH) group of pectin. The identified IR peaks corresponding to the functional groups of pure pectin confirms the presence of pectin molecules on the surface of Ag NPs.

### Colloidal Stability of Silver Nanoparticles in Different Aqueous Environment

**Figure 4:**
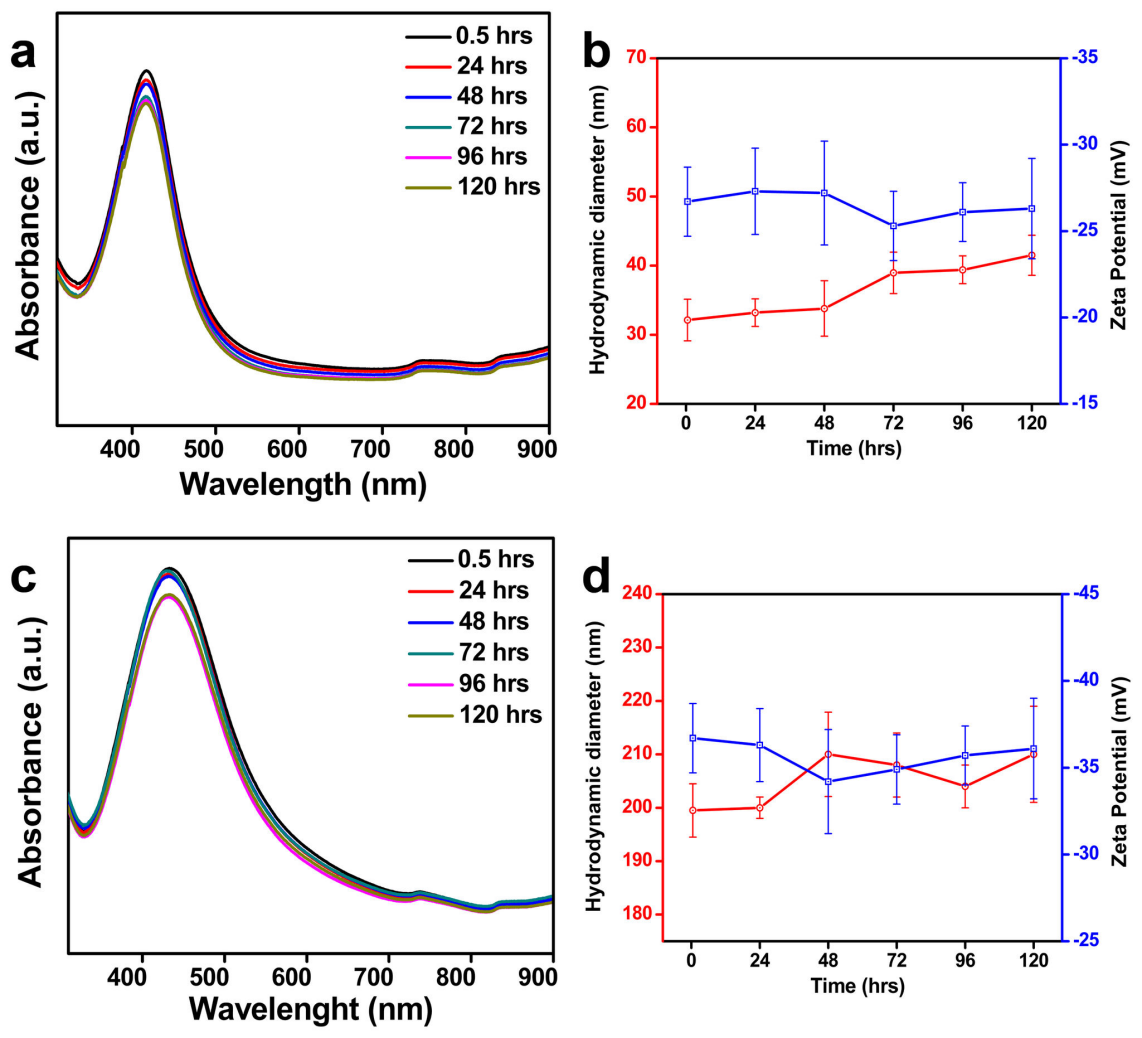
Colloidal stability of bio-silver nanoparticles in water. (a) UV-Vis absorbance spectra, and (b) HDD and ζ potential values of *A. indica* Ag NPs. (c) UV-Vis absorbance spectra and (d) HDD and ζ potential values pectin Ag NPs.

The stability of the NPs were studied in water, electrolyte environment, biological media and in different pH using UV-Vis absorbance spectroscopy by evaluating the changes in their SPR band, and also by studying their changes in HDD and surface charge using zeta measurements. Electrolyte environments - solutions of NaCl and NaNO_3_ with concentrations 50 and 200 mM; biological media -0.1 and 0.25% BSA solution; pH solutions - pH 7 and pH 9, were used to study the aggregation kinetics of the NPs synthesized using *A. indica* leaf extract and pectin solutions for a reasonable time period of 5 days (120 hrs) at temperature close to 25 °C. The UV-Vis absorbance spectra of both Ag NPs suspended in water were very similar and did not show any significant change during the entire study period of 120 hrs (Figure 4a and 4c). Similar to the absorbance spectra, the hydrodynamic size and ζ potential values of the NPs didn’t vary much indicating the stability of particle in water (Figure 4b and 4d).

**Figure 5:**
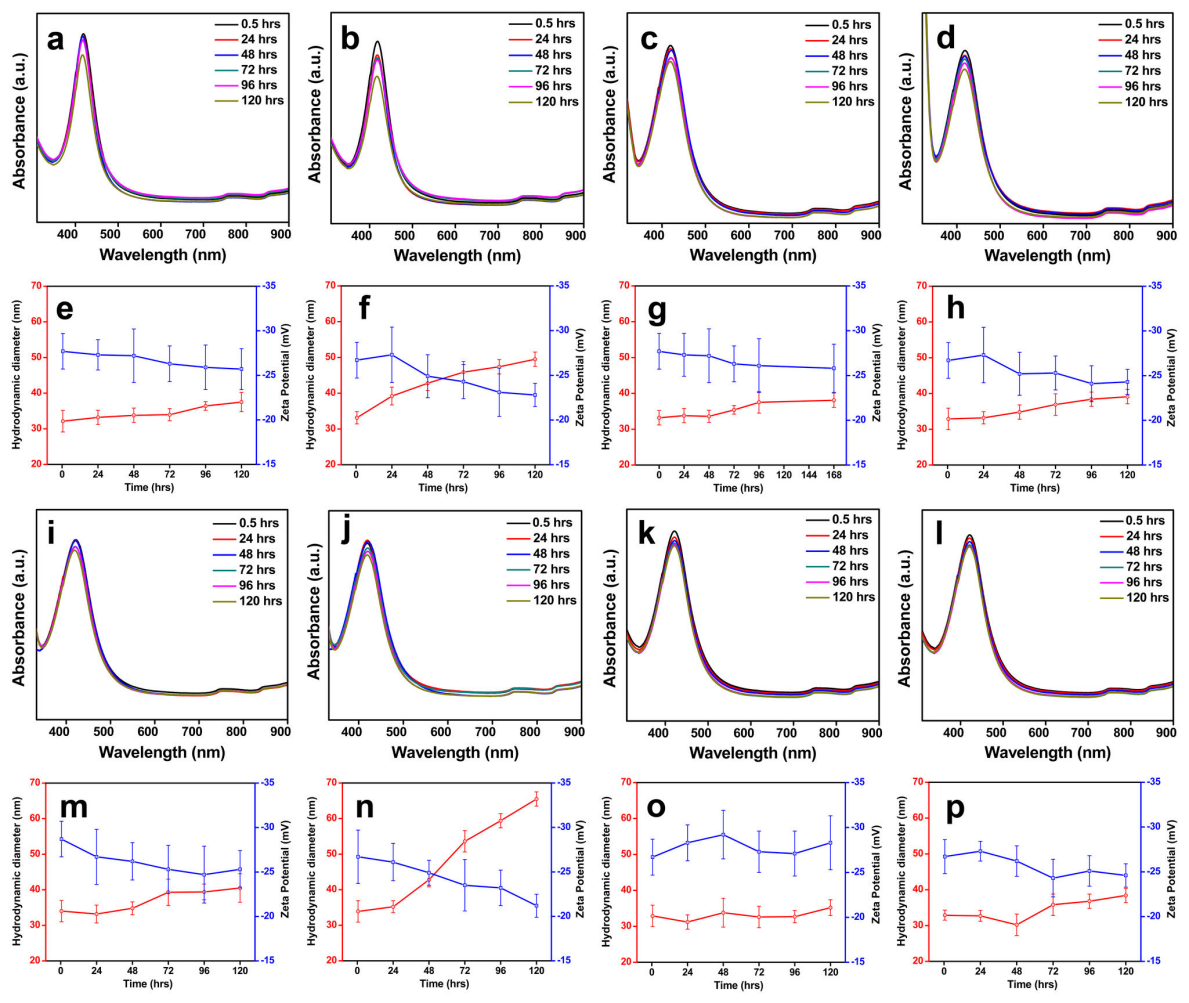
Colloidal stability of *A. indica* Ag NPs in different media. Time dependent UV-Vis absorbance spectra and zeta (HDD and ζ potential value) measurements of *A. indica* Ag NPs dispersed in different media. *A. indica* Ag NPs dispersed in (a-b; e-f) NaCl, (c-d; g-h) NaNO_3_, (i-j; m-n) BSA, (k; o) pH 7 and (l; p) pH 9.

Figure 5 shows the SPR spectra, HDD and ζ values of *A. indica* Ag NPs dispersed in different solutions (NaCl, NaNO_3_, BSA and pH). Observing the SPR spectra of *A. indica* Ag NPs, it is clear that the plasmon bands look nearly similar when dispersed in all the solutions (Figures 5a-d, 5i-l), which indicates that the NPs were stable without any sign of particle aggregation. It was also seen, that there was no significant change in the HDD and ζ potential values of the particles when dispersed in the solutions (Figures 5e-h and 5m-p).

**Figure 6:**
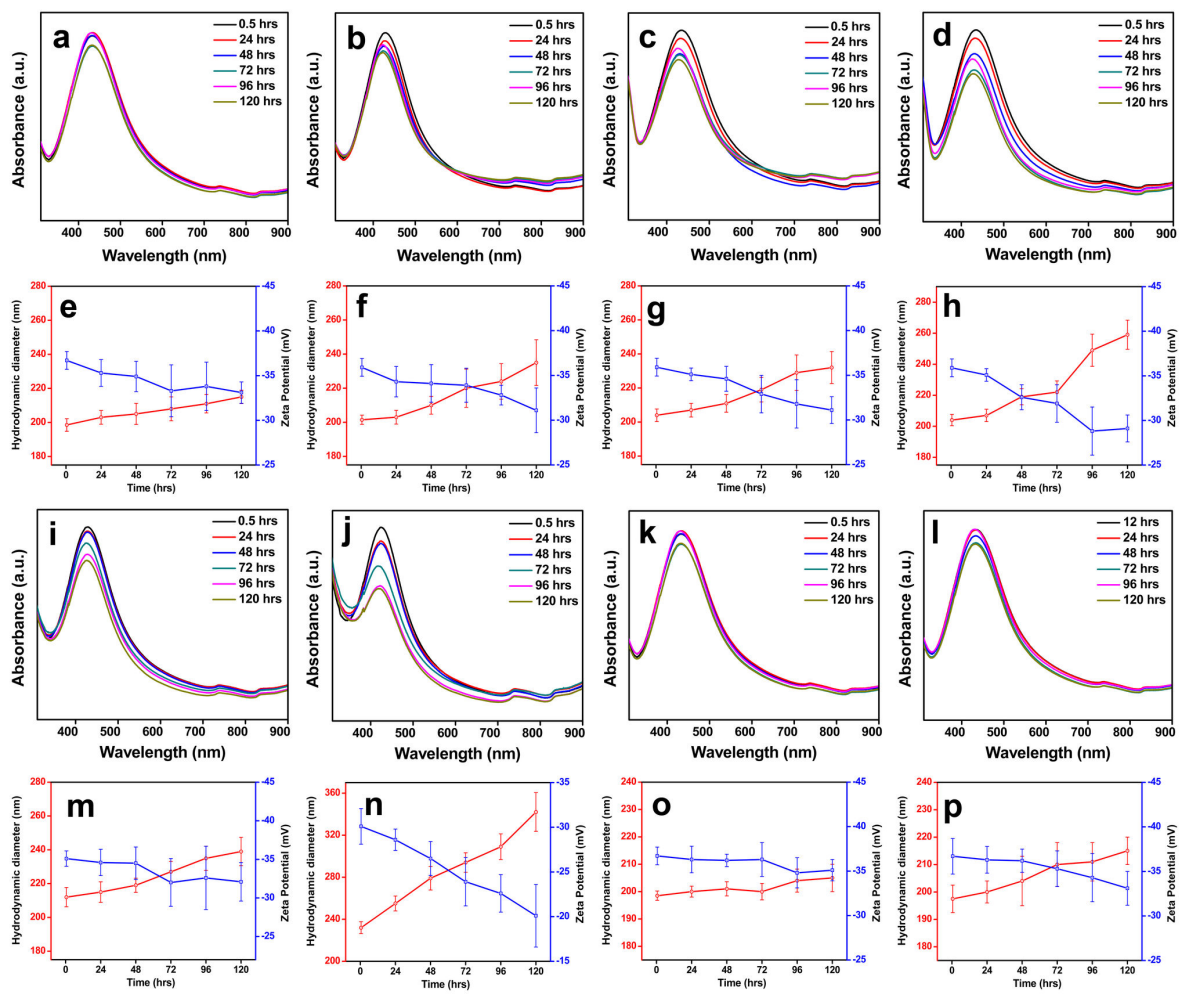
Colloidal stability of pectin Ag NPs in different media. Time dependent UV-Vis absorbance spectra and zeta (HDD and ζ potential value) measurements of pectin Ag NPs dispersed in different media. Pectin Ag NPs dispersed in (a-b; e-f) NaCl, (c-d; g-h) NaNO_3_, (i-j; m-n) BSA, (k; o) pH 7 and (l; p) pH 9.

Pectin Ag NPs dispersed in the NaCl solutions (Figures 6a-b and 6e-f) and in pH 7 and 9 (Figures 6k-l and 6o-p) showed stability for a reasonable time period of 120 hrs with almost identical UV-Vis absorbance spectra and zeta measurements (HDD and ζ potential values). However, with prolonged exposure time the UV-Vis absorbance spectra were not the same when dispersed in NaNO_3_ (Figure 6c-d) and BSA (Figure 6i-j). There was a change in their absorbance intensity as time prolonged when they were dispersed in higher concentration of NaNO_3_ (200 mM) (Figure 6d) and BSA (0.25%) solutions (Figure 6j). Similar to the UV-Vis absorbance spectra, the NPs suspended in 200 mM NaNO_3_ (Figure 6h) and 0.25% BSA (Figure 6n) solutions showed increased hydrodynamic size (above 250 nm at 120 hrs) and low negative ζ values.

### Raman Enhancement Efficiency of Bio-Silver Substrates

**Figure 7:**
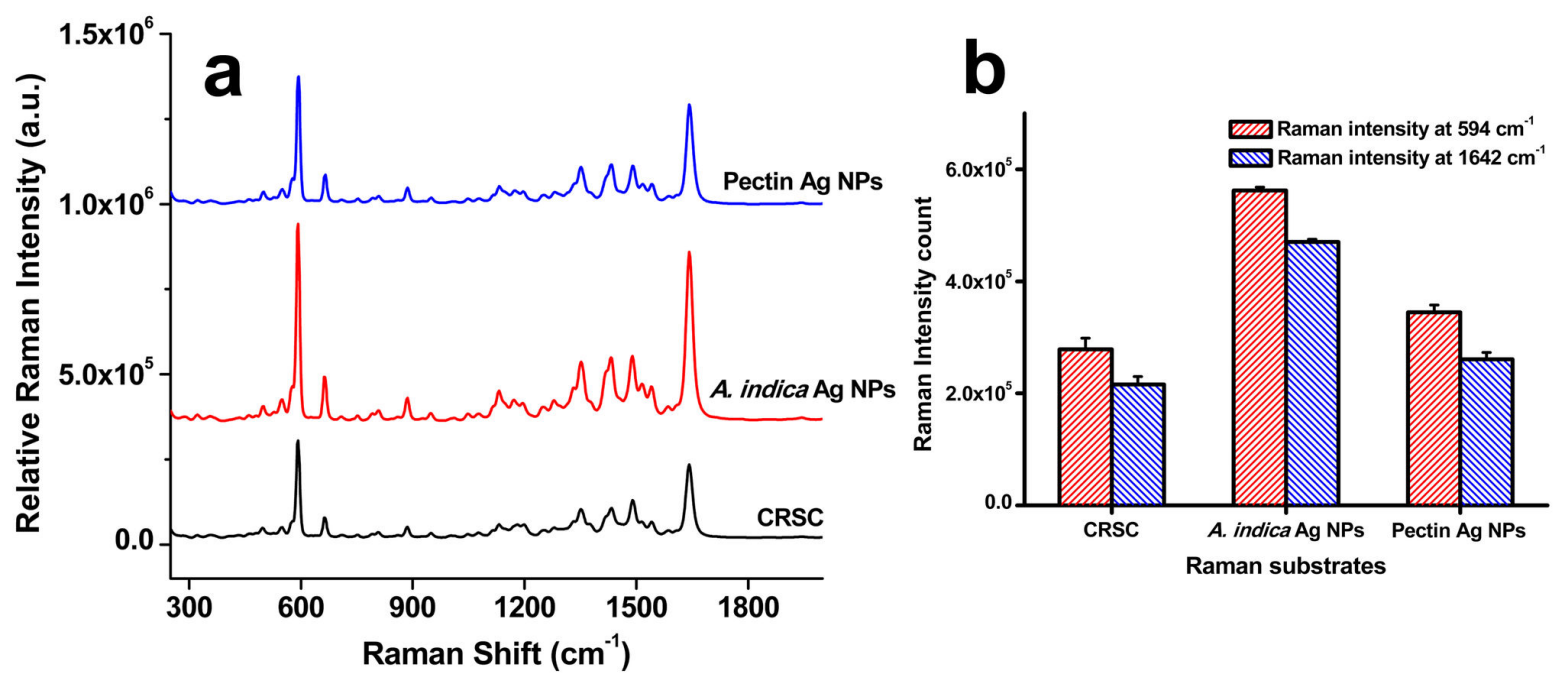
Optical enhancing properties of silver nanoparticles. (a) SERS spectra of NBA adsorbed on *A. indica* Ag NPs, pectin Ag NPs and CRSC substrates. (b) Raman intensity count with respect to Ag substrates at 549 and 1642 cm^-1^.

Optical field enhancing efficiencies of Ag NPs were determined using SERS by enhancing the signals of NBA dye molecules (Figure 7). Included for comparison are standard silver colloids prepared by a citrate reduction method [18]. Clearly the NPs do not give any significant background Raman spectral features (Figure S5). Figure 7a shows the average SERS spectra recorded of 1 × 10^-6^ M NBA solution with each of the silver samples. Also included are the neat NBA Raman spectrum and SERS spectrum recorded when standard citrate reduced silver colloid (CRSC) was probed. All the NBA-Ag NPs showed spectral signals of NBA dye molecule indicating that the substrates are SERS active. However, pure NBA at similar concentration (1 × 10^−6^ M) in water (without Ag substrate) didn’t hardly produce any characteristic spectral signals of NBA (Figure S6). The SERS spectra of NBA consist of several observable Raman peaks, which are intrinsic to chemical bonds seen in NBA [19]. Table 2 details the NBA peaks and their corresponding peak assignments. When the average spectra collected from the Ag substrates was normalized, it was obvious that *A. indica* and Pectin Ag NPs substrates out perform CRSC substrate (Figure 7a). The *A. indica* Ag NPs produced highest SERS signals of NBA followed by pectin Ag NPs and CRSC. Figure 7b shows the Raman intensity counts read at Raman 594 and 1642 cm^-1^. The Raman intensity counts obtained from the *A. indica* Ag NPs were almost two folds higher than that obtained from the CRSC substrate.

**Table 2:** The Raman shifts and peak assignments of Nile blue A.

**Raman shifts (cm^−1^)**				
**CRSC**	***A. indica* Ag NPs**	**Pectin Ag NPs**	**Reported Raman peaks for NBA (cm^−1^)**	**Peak assignments**
592	592	594	590	CCN; CNC
663	662	666		In plane CCC; NCC
885	886	886		
1132	1132	1132	1141	CH bending
1352	1352	1352	1351	Ring stretching
1434	1432	1433		
1490	1490	1490	1492	
1642	1642	1640	1640	

### Bactericidal Activity of Bio-Silver Nanoparticles

**Figure 8:**
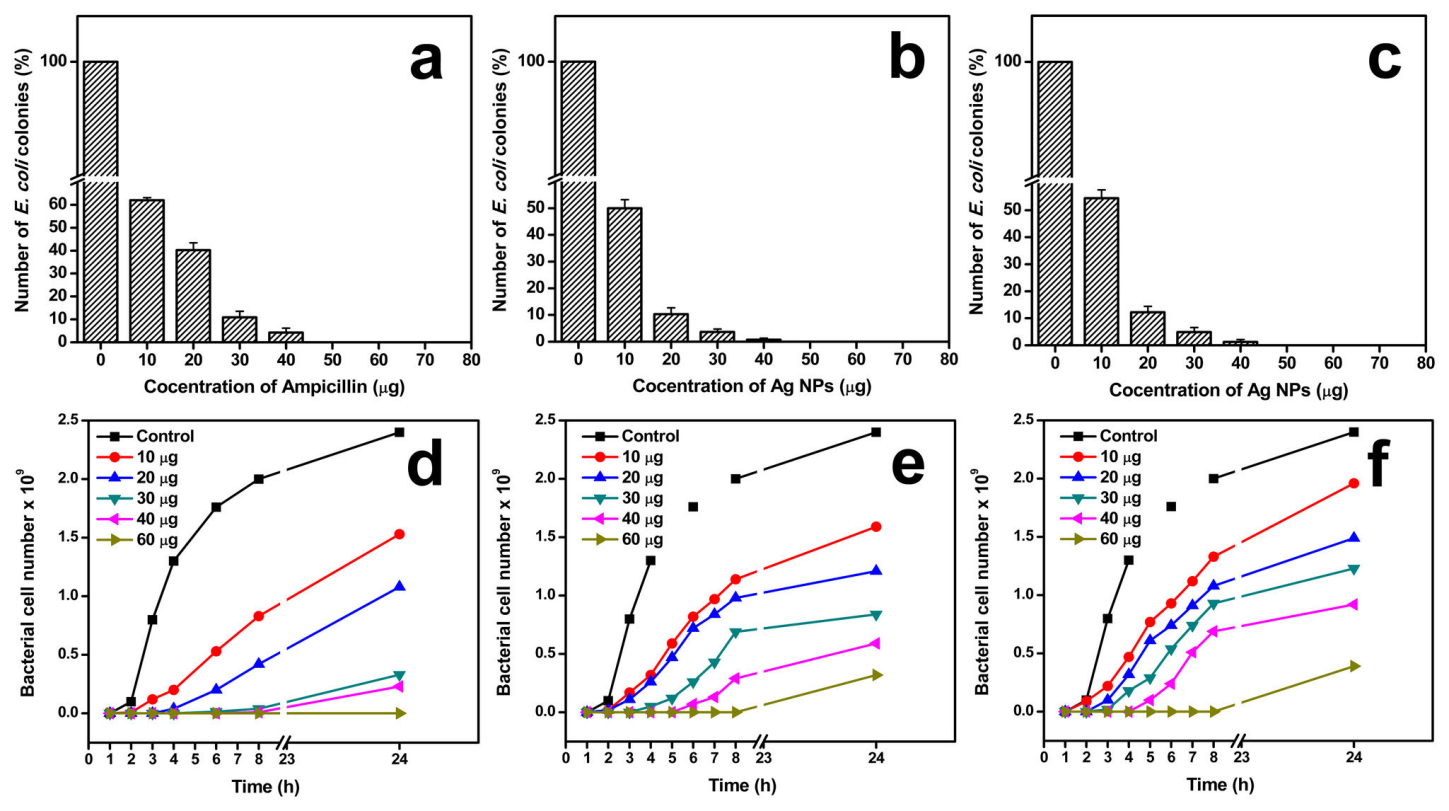
Effect of Ag NPs on bacterial growth. Viability of *E. coli* cells in solid agar and NB liquid media on exposure to antibiotic (ampicillin) and Ag NPs. Percentage histogram plot of the number of *E. coli* colonies as a function of the concentration of (a) ampicillin, (b) *A. indica* Ag NPs and (c) pectin Ag NPs in agar plates. Growth curve of *E. coli* in NB medium supplemented with (d) ampicillin, (e) *A. indica* Ag NPs and (f) pectin Ag NPs.

In order to study the bactericidal activity of the Ag NPs, *E. coli* was grown on nutrient agar plates and NB media supplemented with NPs. Figure 8b and 8c shows the number of bacterial colonies grown on NB agar plates as a function of NPs concentration. The Ag NPs inhibited the bacterial growth of almost 90% when supplemented with 30 µg of Ag NPs in the nutrient agar (Figure 8b and 8c). For both the NPs hardly any colonies of bacteria were found above 40 µg NP concentration, indicating 100% inhibition of growth (Figure 8b and 8c). However, the activity was not the same when identical doses of Ag NPs were studied with the NB liquid medium. Figure 8e and 8f shows the bacterial growth in NB liquid medium supplemented with different concentrations of Ag NPs. Most of the studied concentration of NPs showed bacterial growth and a major difference was the time delay in bacterial growth in the liquid medium. The delay of bacterial growth was seen to be proportionate to the increase in concentration of the NPs (Figure 8e and 8f). On comparison of bactericidal activity of Ag NPs with antibiotic, the ampicillin supplemented plates showed little higher number of bacterial colony, however, concentration above 40 µg completely inhibited the bacterial growth (Figure 8a) as observed with Ag NPs. In liquid culture, concentrations like 10 and 20 µg showed a considerable level of bacterial growth and increase in the concentration further affected the bacterial growth (30 and 40 µg ampicillin supplemented cultures contained < 0.2 × 10^9^ cells after 24 hrs) with complete growth inhibition at the highest studied at a concentration of 60 µg (Figure 8d).

**Figure 9:**
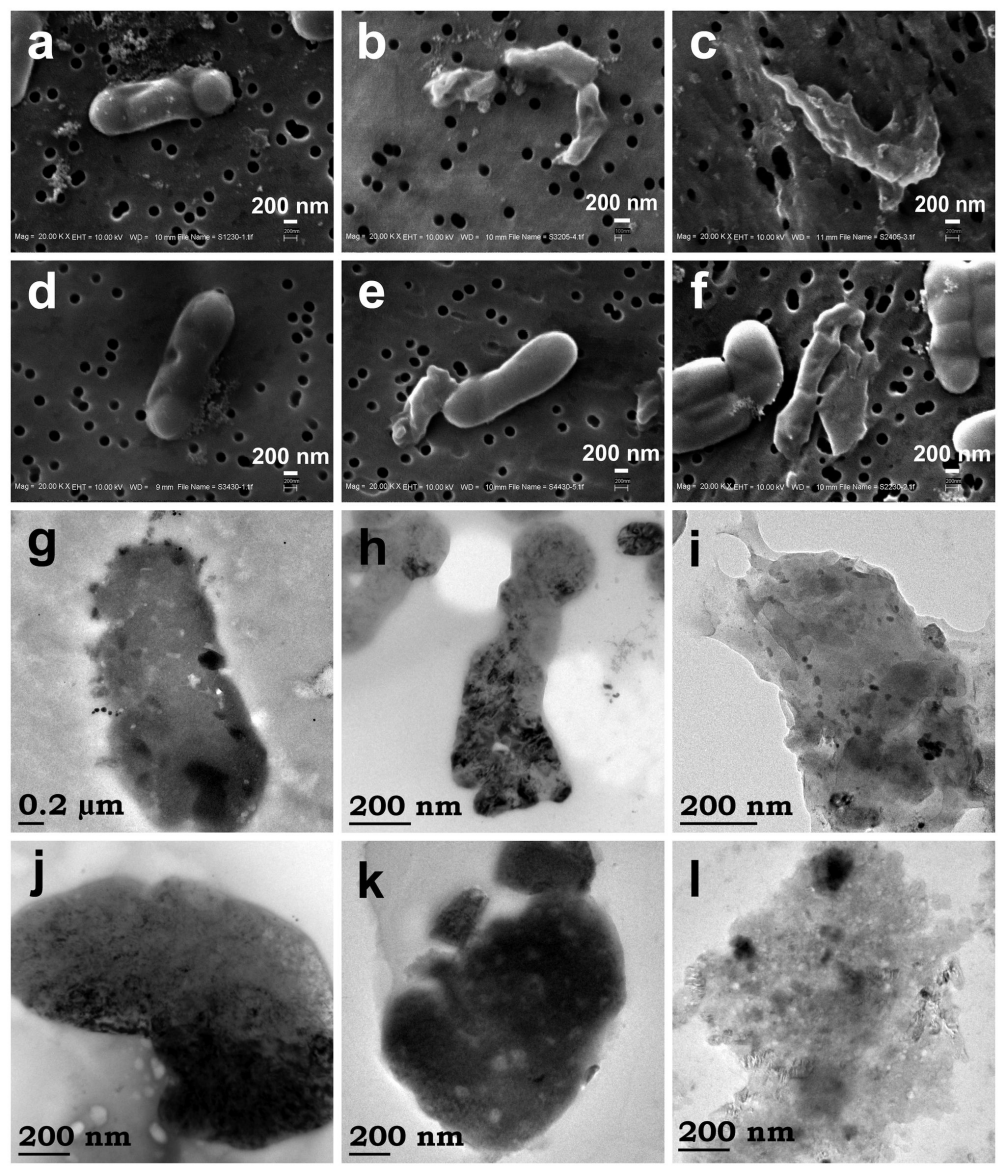
Electron microscopy images of bio-Ag NPs exposed *E. coli* cells. SEM images of *E. coli* cells treated with (a-c) *A. indica* Ag NPs and (d-f) pectin Ag NPs. HRTEM images *E. coli* cells treated with (g-i) *A. indica* Ag NPs and (j-l) pectin Ag NPs.

To study the cellular membrane structure of Ag NPs treated and untreated bacterial cells, electron microscopy (SEM and HRTEM) techniques were employed. Figure 9a-f shows the SEM images of cells treated with *A. indica* Ag NPs (Figure 9a-c) and pectin Ag NPs (Figure 9d-f) (see Figure S7 for SEM image of control bacterium). SEM images clearly indicate the anchoring of Ag NPs on the surface of the bacterial cells making a physical contact (Figure 9a and 9d). It was seen from the images that the cells were completely disintegrated without a definite cell structure evidencing the cell lyses (Figures 9b-c and 9e-f). To get a clear picture on the above parameters such as physical contact of Ag NPs with cellular membrane, membrane rupturing and lyses of cell, HRTEM images of Ag NPs treated *E. coli* were acquired. Physical interaction of the Ag NPs to the *E. coli* cellular membrane was evident with the HRTEM images (Figure 9g and 9j). The structural damage (Figure 9h and 9k) and complete disintegration (or degradation) of the cellular membrane structure were also observed (Figure 9i and 9l).

## Discussion

The use of plant-derived materials, in particular, the leaf extract has been extensively studied over the past years in synthesis of Ag NPs. The phytochemicals present in plant extracts such as water-soluble proteins, polyphenols, and sugars have been reported to play a significant role in reducing and stabilizing the NPs [20-22]. The presence of the above phytochemicals in the leaf extract have been determined using UV-Vis absorbance spectroscopy (Figure S3a) and through standard biochemical estimations (Figure S3b). However, it is obvious that determining the specific role of these phytochemicals is ruled-out, since most of them have dual function during synthesis. For example, proteins and sugars can actively participate in both reduction of metal ions and in stabilization of the NP formed [20]. The FTIR spectra of *A. indica* Ag NPs (Figure 3a) indicate the presence of functional groups corresponding to polyphenols, amides and carbohydrates on the NP surface proving that these molecules contribute to the stability of NPs. *A. indica* phytochemicals capping the NPs makes them colloidally stable in different media such as electrolyte solutions (NaCl; NaNO_3_), biological solution (BSA) and in pH solutions (pH 7 and pH 9). The UV-Vis spectra and zeta measurements remained almost same for NPs suspended in all media throughout the study (Figure 5). It is conceivable that the phytochemicals in *A. indica* leaf extract provide a strong coating on the surface of the NPs resulting in electrostatic repulsion between the NPs in the different aqueous environments leading to efficient stability.

Polymers are known to offer control over reduction rate, enabling NPs of different shapes and sizes, and also can provide an inelastic stability to NPs preventing their aggregation. Pectin used in the study is a non-toxic; naturally occurring plant polysaccharide (biopolymer) present in the peels of apple, citrus fruits and also found in some vegetables [23,24]. Structurally pectin is a linear polysaccharide consisting of D-galacturonic acid units joined in chain by means of α-(1-4) glycosidic linkage (Figure 1b) [25,26]. The presence of functional groups such as carboxyl and hydroxyl groups makes pectin an ideal molecule for complexing with various metals [27,28]. The presence of pectin molecules on the surface of the NPs is evident by the FTIR spectrum (Figure 3b). It was observed from the FTIR spectra that the intense peak at 1744 cm^−1^ for pure pectin (COOH group of pectin) is obviously weaker for the pectin Ag NPs (Figure 3b and Figure S4). The above changes in the FTIR pattern may indicate the participation of carboxyl group in formation of the NPs. The pectin-Ag NPs showed excellent level of colloidal stability in the electrolyte solutions (NaCl and NaNO_3_ [50mM]), in different pH solutions (pH 7 and 9) and in lower percentage of biological solution (BSA) (Figure 6). However, when pectin Ag NPs were dispersed in higher concentration of NaNO_3_ (200 mM) and BSA (0.25%) solutions, decrease in their SPR band intensities were observed. Difference in the absorption intensities of NPs can be due to the sedimentation of colloidal particles at higher strength of NaNO_3_ and BSA solutions [29,30], which might have influenced the availability of free NPs in the suspension media resulting in decrease in absorbance intensity.

A careful survey of literature indicates that in most of the NPs synthesized using plant-derived materials, research has stopped at the level of basic material characterization without exploring its application. Here, the NPs were studied for optical field enhancing property using SERS by enhancing the signals of NBA using the *A. indica* Ag NPs and pectin Ag NPs as the substrates. Additionally, antibacterial property was determined by treating *E. coli* with bio-NPs. In determining the SERS efficiency of the Ag NPs substrate, *A. indica* Ag NPs were observed to enhance the signals of NBA more than that of the pectin NPs. As reported elsewhere, the SERS efficiency is dependent on the surface charge and efficient adsorption of the analyte molecule on the surface of the NPs [31,32]. The higher negative surface charge of the pectin Ag NPs (ζ potential value of -36.2 mV) may prevent the particle aggregation and also influence the adsorption of analyte on the surface of the particles, hence affecting SERS enhancement [31,32]. Figure S8 shows the plot matching the SERS intensity count at Raman shift 594 cm^−1^ with ζ potential values of plant Ag substrates, clearly indicating dependable SERS activity with NP surface charge.

All studied test compounds (antibiotic and Ag NPs) showed almost similar level of bactericidal activity on agar plates. Concentrations above 40 µg completely inhibited the bacterial growth. However, in the liquid medium the bacterial growth was observed in all the studied NP concentrations, the only difference is the initial growth delay of the bacterial cells, which increased as the concentration of the NPs increased in the medium. On other hand, at concentration above 30 µg of ampicillin the bacterial growth was greatly affected with complete inhibition even after 24 hrs when supplemented with 60 µg of ampicillin. It was explained elsewhere, that the NPs in liquid medium only delays the bacterial growth [12,13]. It has been stated that, as the time prolongs, the concentration of the Ag NPs in the medium decreases due to the interaction of the NPs with intracellular components from the deceased bacterial cells causing particle coagulation, and allowing resumption of the bacterial growth [12,13]. Even though the pectin NPs possessed smaller grain size and higher negative ζ potential value (-36.2 mV), it displayed almost similar *E. coli* cell growth pattern in the liquid medium as that of *A. indica* Ag NPs. As observed by the behavior of pectin Ag NPs in BSA solution (Figure 6j and 6n), the pectin Ag NPs might have interacted with the intracellular components (peptides) from the lysed cells, thus minimizing the dispersion state of NPs in the NB media leading to ineffective antibacterial activity.

Although the exact mechanism of bactericidal action of Ag NPs has not been fully explained, the physical damage of the bacterial membrane caused by the NP interaction may lead to cell death. However, the mode of interaction (anchoring) between *E. coli* cell surface and NP (through any specific receptors or molecular species in cell wall) is not yet resolved fully [13,33]. In a recent study, effective anchoring of Ag NPs on *E. coli* cell surface was stated due to the adsorbed organic molecule on the NP surface from the guava leaf broth during synthesis, which aided in better interaction with the cell surface when compared to the chemically synthesized Ag NPs [33]. Polysaccharides (exopolysaccharide) surfaced Ag NPs are also reported for a higher adherence towards bacterial surface to provide an efficient bactericidal activity [34]. As stated in above literatures, the organic (phytochemicals) coat on *A. indica* Ag NPs and polysaccharide (pectin) coat on pectin Ag NPs might have been aided in their better interaction with the bacterial cell wall [33,34]. A good physical interaction between the NP and the bacterial cell wall is vital for the cell damage [35]. Physical interaction of the NPs with the cellular membrane may cause physical damage (through pitting) (Figures 9a, 9d, 9g and 9j) that ultimately leads to cellular death or pass through membrane [36,37]. Similarly Sondi and Sondi [13] reported that the Ag NPs causes ‘pits’ in the cellular membrane of bacteria, leading to increased membrane permeability and finally, causing cell death. Increase in the membrane permeability was observed by Stoimenov and co-workers [38] when *E. coli* cells were treated with highly reactive metal oxide. Increase in the permeability affects the bacterial cells to regulate transport through the plasma membrane and, finally, leading to cell death [38]. *E. coli* membrane is known to consist of lipopolysaccharids (LPS) in the outer membrane which serves as an effective permeability barrier [39,40]. The interaction of NPs with the membrane may affect the LPS and other membrane proteins, causing change in the membrane permeability (or degradation of membrane structure) (Figure 9) [13,33,34,41].

## Conclusions

We herein have presented a facile green method for synthesis of Ag NPs using leaf extracts of *A. indica* and biopolymer pectin from apple peel. Both the NPs showed a considerable level of stability in most of the studied media for a reasonable time period of 120 hrs. On determining the SERS activity, both the studied Ag NPs displayed elegant SERS signals of Raman analyte NBA describing their SERS activity. SERS signals exhibited by the studied Ag NPs substrates were higher than that of the routinely synthesized CRSC; in particular the Ag NPs synthesized using *A. indica* leaf extract showed the highest SERS efficiency. On studying the bactericidal activity of the Ag NPs, it was found that above 40 µg of Ag NPs concentration in agar plates completely inhibited bacterial growth. However, in the liquid culture, the NPs caused a growth delay and the delay time increased in proportion to the increase in concentration of the Ag NPs in the media. The electron microscopy images evidenced the physical interaction between the NPs and cellular membrane and also the damages caused by NP to the bacterial cell wall integrity. These stable green Ag NPs can be applied in biology for detection based on SERS and in bacterial infection control.

## Supporting Information

Figure S1 **Crystalline phase of bio-Ag NPs.** Powder XRD pattern of Ag NPs synthesized using *A. indica* plant leaf extract and biopolymer pectin.
(TIF)

Figure S2 **Elemental analysis of the bio-Ag NPs.** EDS of (a) *A. indica* and (b) pectin Ag NPs.
(TIF)

Figure S3 **Characterization of leaf extracts.** (a) UV-Vis absorbance spectrum of leaf extract. Strong absorption band at ~ 272 nm is assigned to the aromatic side group of amino acid residue of protein. The accompanying peak at absorbance ~ 325 nm could be arisen from the water-soluble phenolic compounds in the extract. (b) Concentration of total soluble proteins (TSP), total sugars and total phenolic content (TPC) in final leaf filtrate.
(TIF)

Figure S4 **Chemical composition of pectin.** FTIR spectrum of pure pectin.
(TIF)

Figure S5 **Background Raman spectra of Ag NPs.** Raman spectra of blank silver nanoparticles (without Raman analyte molecule NBA).
(TIF)

Figure S6 **Neat Raman spectrum of NBA dye molecule.** Raman spectrum of 1 × 10^−6^ M Nile Blue A in water without silver substrate.
(TIF)

Figure S7 **Electron microscopy photo of *E. coli*.** SEM image of Ag NPs untreated *E. coli* cells.
(TIF)

Figure S8 **Comparison of zeta potential values of bio-Ag NPs with their respective SERS intensities.** Plot of SERS intensity count at 594 cm^−1^ versus zeta potential values of Ag NPs.
(TIF)
