# Supplementary figures and images for: Differently Environment Stable Bio-Silver Nanoparticles: Study on Their Optical Enhancing and Antibacterial Properties
Source: PLoS One. 2013 Oct 9;8(10):e77043. doi: 10.1371/journal.pone.0077043 (PMC3793943; doi:10.1371/journal.pone.0077043)

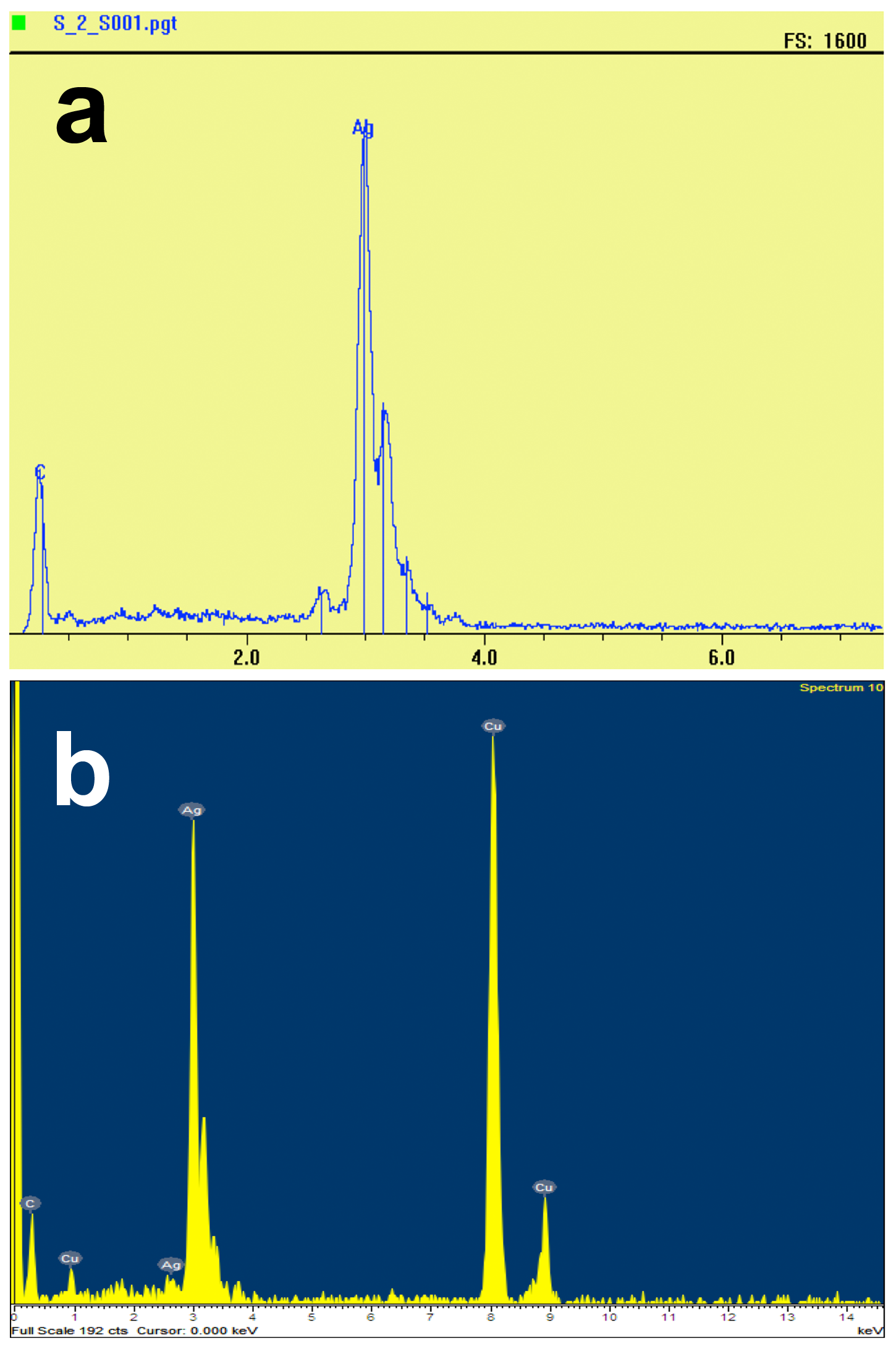

Supplement: Figure S2 — Elemental analysis of the bio-Ag NPs. EDS of (a) A. indica and (b) pectin Ag NPs. (TIF) [file pone.0077043.s002.tif]

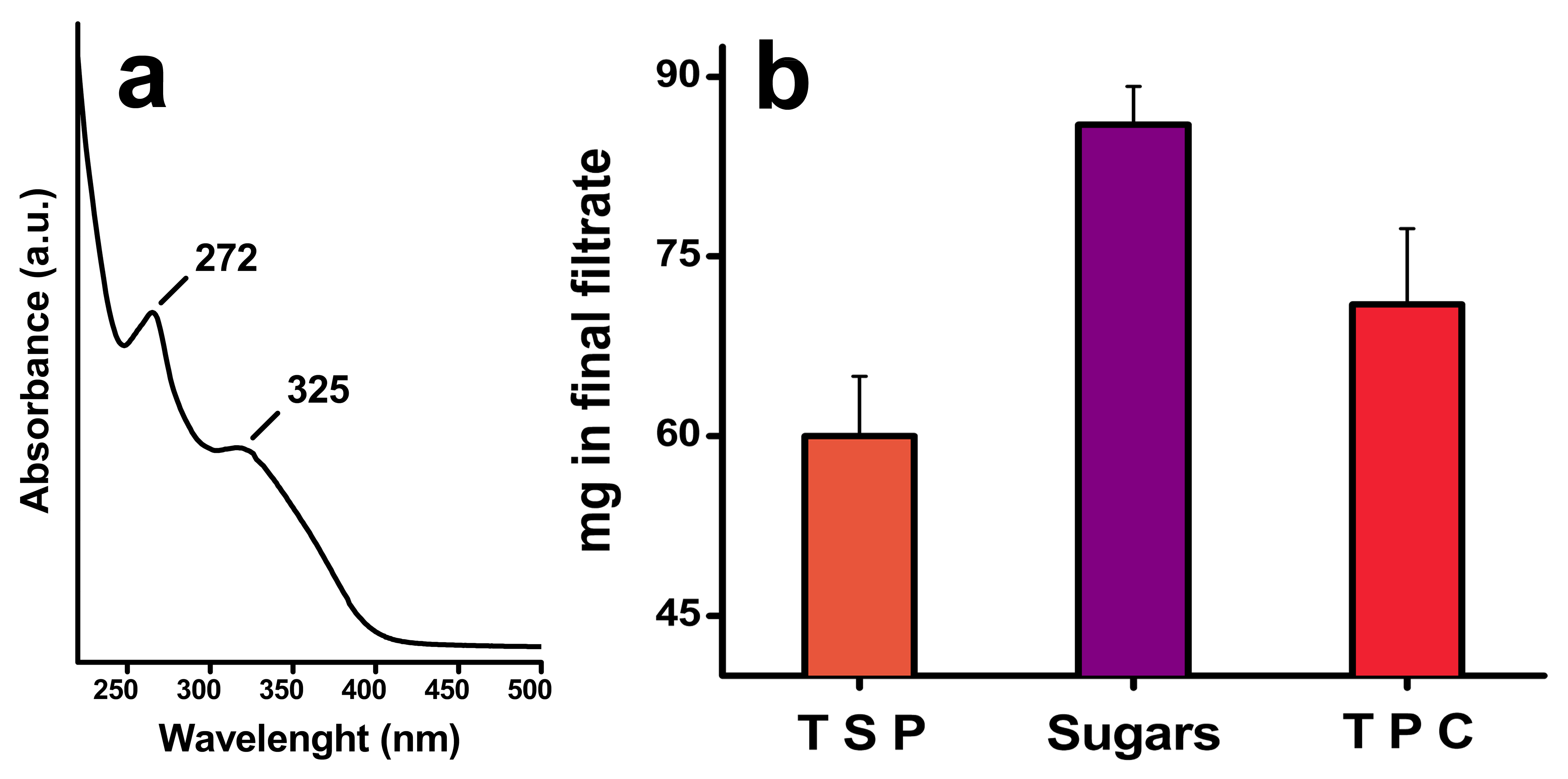

Supplement: Figure S3 — Characterization of leaf extracts. (a) UV-Vis absorbance spectrum of leaf extract. Strong absorption band at ~ 272 nm is assigned to the aromatic side group of amino acid residue of protein. The accompanying peak at absorbance ~ 325 nm could be arisen from the water-soluble phenolic compounds in the extract. (b) Concentration of total soluble proteins (TSP), total sugars and total phenolic content (TPC) in final leaf filtrate. (TIF) [file pone.0077043.s003.tif]

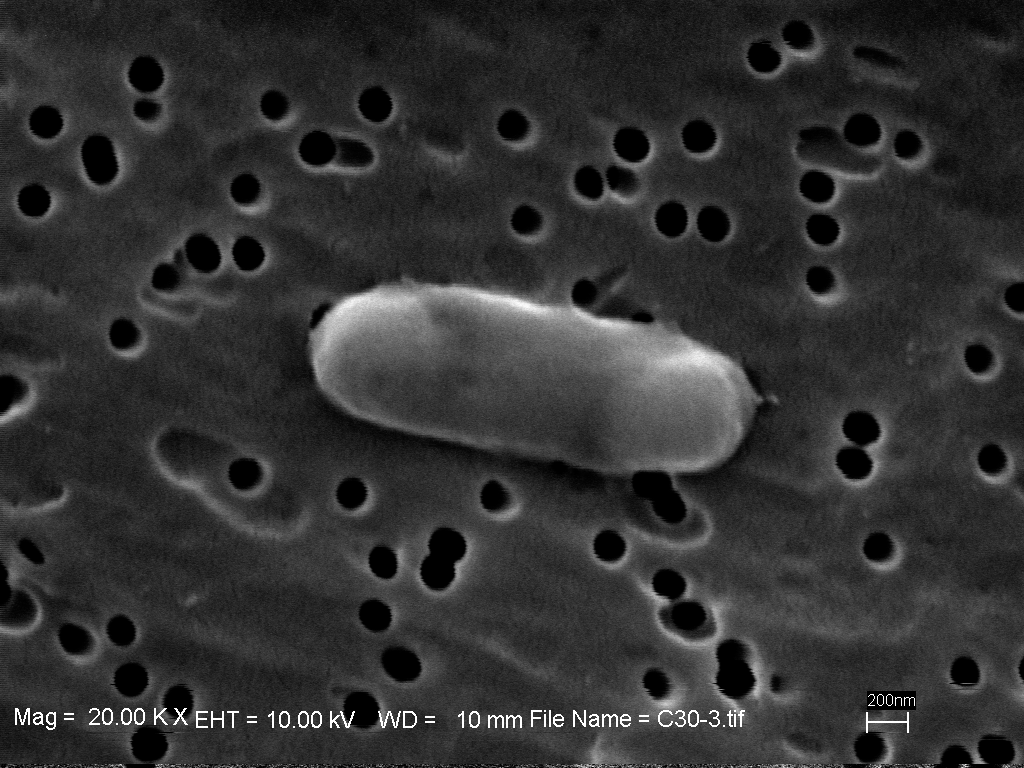

Supplement: Figure S7 — Electron microscopy photo of E. coli. SEM image of Ag NPs untreated E. coli cells. (TIF) [file pone.0077043.s007.tif]
